# Complete Genome Sequences for Three Chromosomes of the *Burkholderia stabilis* Type Strain (ATCC BAA-67)

**DOI:** 10.1128/genomeA.01294-16

**Published:** 2016-11-17

**Authors:** Julia V. Bugrysheva, Blake Cherney, David Sue, Andrew B. Conley, Lori A. Rowe, Kristen M. Knipe, Michael A. Frace, Vladimir N. Loparev, Julie R. Avila, Kevin Anderson, David R. Hodge, Segaran P. Pillai, Linda M. Weigel

**Affiliations:** a National Center for Emerging and Zoonotic Infectious Diseases, Office of Infectious Diseases, Centers for Disease Control and Prevention, Atlanta, Georgia, USA; b IHRC, Atlanta, Georgia, USA; c Physical and Life Sciences Directorate, Lawrence Livermore National Laboratory, Livermore, California, USA; d Science and Technology Directorate, Department of Homeland Security, Washington, District of Columbia, USA; e Office of Laboratory Science and Safety, Food and Drug Administration, Silver Spring, Maryland, USA

## Abstract

We report here the complete annotated genome sequence of the *Burkholderia stabilis* type strain ATCC BAA-67. There were three circular chromosomes with a combined size of 8,527,947 bp and G+C composition of 66.4%. These characteristics closely resemble the genomes of other sequenced members of the *Burkholderia cepacia* complex.

## GENOME ANNOUNCEMENT

The *Burkholderia stabilis* strain ATCC BAA-67, which is referred to in other culture collections as LMG 14294, CCUG 34168, CIP 106845, or NCTC 13011, was originally isolated in 1993 from the sputum from a cystic fibrosis patient in Leuven, Belgium (1). The *B. stabilis* species belongs to the *Burkholderia cepacia* complex (Bcc) and can be distinguished from other members of this complex by its biochemical and physiological properties (1). ATCC BAA-67 is the type strain for the *B. stabilis* species.

Genomic DNA for sequence analysis was extracted using the MasterPure complete DNA and RNA purification kit (Epicentre), according to the manufacturer’s DNA purification protocol for cell samples. The 20-kb libraries were generated with the Pacific Biosciences SMRTbell DNA template preparation kit. Sequence analysis was performed with the DNA/polymerase binding kit P6 version 2 and a PacBio RSII sequencer with two single-molecule real-time cells using C4 version 2 chemistry and 360-min movies.

Sequence reads were assembled *de novo* using RS_HGAP Assembly.3 in the SMRT Analysis 2.3.0 portal, resulting in three complete circularized contigs that correspond to three chromosomes for this strain. The average depth of coverage was >105× for all three chromosomes. This assembly was verified using whole-genome mapping (Argus; OpGen) with the BamHI restriction enzyme (New England BioLabs).

The BAA-67 chromosome sizes were 3,886,092 bp, 3,318,880 bp, and 1,322,975 bp. G+C composition values were 66.3%, 66.9%, and 65.5% for each chromosome, respectively. The number of chromosomes, their lengths, and the high G+C content are similar to previously sequenced Bcc strains (see references 2 and 3 or Bcc taxid: 87882 on http://www.ncbi.nlm.nih.gov/genome/browse/).

The genome of BAA-67 was annotated with the Prokaryotic Genomes Annotation Pipeline at NCBI (NCBI_PGAP). It was predicted to contain 7,425 coding genes, 6 rRNA operons, and 68 tRNAs. Chromosome 1, the largest, contained three intact rRNA operons and one operon, with the 23S rRNA gene broken by the insertion of a transposase gene. Chromosomes 2 and 3 each contained one rRNA operon with a complete set of 16S, 23S, and 5S rRNA genes. Although the third and smallest chromosome carries rRNA genes and multiple unique enzymes, it may be nonessential for growth *in vitro*, since Bcc bacteria, including *B. stabilis* species, could be cured of this replicon (4, 5). The third chromosome has been described as a virulence plasmid, because its loss is associated with loss of virulence in several infection models (4, 5).

Previously reported pulsed-field gel electrophoresis typing experiments indicate that the genomes of different strains within *B. stabilis* species are conserved (1), suggesting that the genome of the strain ATCC BAA-67, which is the first completely sequenced genome for *B. stabilis*, is a good reference model for studies of other strains of this species.

### Accession number(s).

The finished annotated sequences of the three chromosomes of *Burkholderia stabilis* ATCC BAA-67 have been deposited in NCBI GenBank under the accession numbers CP016442, CP016443, and CP016444. The sequence reads were uploaded to the NCBI SRA under the accession number SRP079204.
